# Tangential Versus Segmental Portomesenteric Venous Resection During Pancreatoduodenectomy for Pancreatic Cancer: An International Multicenter Cohort Study on Surgical and Oncological Outcome

**DOI:** 10.1097/SLA.0000000000006638

**Published:** 2025-01-22

**Authors:** Thomas F. Stoop, Adrienne Molnár, Leonard W.F. Seelen, Toshitaka Sugawara, Jacobien C.M. Scheepens, Mahsoem Ali, Ammar A. Javed, Asif Halimi, Atsushi Oba, Bas Groot Koerkamp, Bodil Andersson, Caroline Williamsson, Christopher L. Wolfgang, Daisuke Ban, Ernesto Sparrelid, Freek Daams, Geert Kazemier, Hjalmar C. van Santvoort, Ingmar F. Rompen, I. Quintus Molenaar, Joseph R. Habib, Lysanne P.M. Beuk, Niek J. Geerdink, Roeland F. de Wilde, Olivier R. Busch, Oskar Swartling, Paulina Bereza-Carlson, Poya Ghorbani, Reeve L. Kruize, Richard D. Schulick, Salvador Rodriguez Franco, Tatsunori Miyata, Oskar Franklin, Yosuke Inoue, Marc G. Besselink, Marco Del Chiaro

**Affiliations:** * Amsterdam UMC, location University of Amsterdam, Department of Surgery,Amsterdam, The Netherlands; † Cancer Center Amsterdam, Amsterdam, The Netherlands; ‡ Department of Surgery, Division of Surgical Oncology, University of Colorado Anschutz Medical Campus, Aurora, CO; § Department of Clinical Science, Intervention and Technology, Division of Surgery, Karolinska Institutet at Karolinska University Hospital, Stockholm, Sweden; ∥ Department of Diagnostics and Intervention, Surgery, Umeå University, Umeå, Sweden; ¶ Department of Surgery, Regional Academic Cancer Center Utrecht, University Medical Center Utrecht/St. Antonius Hospital Nieuwegein, Utrecht & Nieuwegein, The Netherlands; # Department of Hepatobiliary and Pancreatic Surgery, Graduate School of Medicine, Institute of Science Tokyo, Tokyo, Japan; ** Amsterdam UMC, locationVrije University, Department of Surgery,Amsterdam, The Netherlands; †† Department of Surgery, Division of Surgical Oncology, New York University Medical Center, New York City, NY; ‡‡ Division of Hepato-biliary-pancreatic Surgery, Cancer Institute Hospital, Japanese Foundation for Cancer Research, Tokyo, Japan; §§ Department of Surgery, Erasmus MC Cancer Institute, University Medical Center Rotterdam, Rotterdam, The Netherlands; ∥∥ Department of Clinical Sciences Lund, Surgery, Lund University and Skåne University Hospital, Lund, Sweden

**Keywords:** pancreatoduodenectomy, pancreatic cancer, venous resection, tangential, segmental, survival

## Abstract

**Objective:**

To investigate whether tangential versus segmental portomesenteric venous resection (PVR) impacts surgical and oncological outcomes in patients undergoing pancreatoduodenectomy for pancreatic cancer with portomesenteric vein (PMV) involvement.

**Background:**

Current comparative studies on tangential versus segmental PVR as part of pancreatoduodenectomy for pancreatic cancer include all degrees of PMV involvement, including cases where tangential PVR may not be a feasible approach, limiting the clinical applicability.

**Methods:**

An international retrospective study in 10 centers from 5 countries, including all consecutive patients after pancreatoduodenectomy with PVR for pancreatic cancer with ≤180 degrees PMV involvement on cross-sectional imaging at diagnosis (2014–2020). Cox and logistic regression analyses were performed to investigate the association of tangential versus segmental PVR with overall survival (OS) from surgery, recurrence-free survival (RFS), locoregional recurrence, and in-hospital/30-day major morbidity, adjusting for potential confounders.

**Results:**

Overall, 357 patients who underwent pancreatoduodenectomy with PVR were included (42% tangential PVR, 58% segmental PVR). The adjusted risk for in-hospital/30-day major morbidity was 23% (95% CI, 17–32) after tangential and 23% (95% CI, 17–30) after segmental PVR (*P*=0.98). After adjusting for confounders, PVR type was not associated with OS [HR=0.94 (95% CI, 0.69–1.30)], RFS [HR=0.94 (95% CI, 0.69–1.28)], and locoregional recurrence [OR=0.76 (95% CI, 0.40–1.46)].

**Conclusions:**

In patients undergoing pancreatoduodenectomy for pancreatic cancer with ≤180 degrees PMV involvement, the type of PVR (ie, tangential vs segmental) was not associated with differences in surgical and oncological outcome. This suggests that if both procedures are technically feasible, surgeons can choose the type of PVR based on their preference.

Surgery combined with chemotherapy is the cornerstone of treatment of pancreatic ductal adenocarcinoma (hereafter: *pancreatic cancer*).^1,2^ In about 70% of these patients, the tumor is located in the pancreatic head often involving the portomesenteric vein (PMV; ie, portal vein, confluence, and/or superior mesenteric vein).^1,3^ In case of PMV involvement, a pancreatoduodenectomy combined with portomesenteric venous resection (PVR) is frequently necessary and has become a standard procedure for pancreatic surgeons over the last decades.^4–6^

Different types of PVR can be performed and have been classified by the International Study Group of Pancreatic Surgery (ISGPS) as tangential resection with primary closure (type 1), tangential resection with patch reconstruction (type 2), segmental resection with end-to-end reconstruction (type 3), and segmental resection reconstructed by using an interposition graft (type 4).^7^ The decision to perform either a tangential (type 1–2) or segmental (type 3–4) PVR is not only influenced by the extent of the venous tumor involvement, but also by the surgeons’ preference and experience.^8,9^

A recent nationwide observational study from the Netherlands found that segmental PVR was associated with an increased likelihood to develop major morbidity and shorter overall survival (OS),^10^ but this is contradictory to results from other studies.^11,12^ However, other series are hampered by the fact that pancreatic tumors with any extent of PMV involvement were included, including cases in which a tangential resection would technically not be an option at all.^13,14^ Moreover, the inclusion of a wide spectrum of PMV involvement may have influenced R status and OS. Therefore, it remains unknown in patients who undergo pancreatoduodenectomy for pancreatic cancer with limited PMV involvement whether the type of PVR in patients impacts the surgical and/or oncological outcomes.

Hypothetically, segmental PVR increases the chance for an R0 resection compared with a tangential PVR, which in return may lead to a lower rate of locoregional recurrence and, therefore, prolonged OS.^15–18^ This study aimed to investigate the impact of the type of PVR (ie, tangential vs segmental) on the surgical and oncological outcome after pancreatoduodenectomy in patients with only ≤180 degrees involvement of the PMV.

## METHODS

This retrospective, observational, multicenter cohort study is reported in accordance with the Strengthening the Reporting of Observational Studies in Epidemiology (STROBE) guideline.^19^ The study procedures were reviewed and approved by the Colorado Multiple Institutional Review Board (COMIRB) (#19-2972) at the University of Colorado, the ethical committee from the Amsterdam UMC (#2023.0520), and the Swedish Ethical Review Authority (2024-02309-01). The need for informed consent was waived because of the retrospective nature of this study.

### Study Design and Patients

All consecutive patients (≥18 y of age) who underwent PVR during pancreatoduodenectomy for pancreatic ductal adenocarcinoma with ≤180 degrees involvement of the PMV on cross-sectional imaging at the time of diagnosis (2014–2020) were included.

Exclusion criteria comprised (1) PMV occlusion, partial/complete thrombosis, and/or lumen narrowing on cross-sectional imaging at the time of diagnosis; (2) any arterial involvement with the superior mesenteric artery (SMA), celiac axis, and/or hepatic artery on cross-sectional imaging at the time of diagnosis; (3) extended resection(s) other than PVR (ie, multivisceral, arterial, and/or venous resection[s])^20^; (4) R2 resection; (5) metastatic disease before surgery or based on final histopathology. Of note, resection of the inferior mesenteric vein was not used as an exclusion criterion.

The comparison of interest is outlined using the PICO framework^21^: [P] patients who underwent PVR during pancreatoduodenectomy for pancreatic cancer with ≤180 degrees involvement of the portomesenteric venous axis on cross-sectional imaging at the time of diagnosis, comparing [I] PVR type 3 to 4 (ie, segmental resection) with [C] PVR type 1 to 2 (ie, tangential resection). [O] The primary outcome of interest was OS measured from surgery. Secondary endpoints were in-hospital/30-day major morbidity, portomesenteric venous thrombosis, R0 status, 90-day mortality, recurrence-free survival (RFS), and locoregional recurrence.

### Definitions

Patient performance status at the time of surgery was defined using the Eastern Cooperative Oncology Group (ECOG) performance status.^22^ The 8th edition of the TNM classification was used for both clinical and pathologic disease staging.^23^ Radiologic response following neoadjuvant therapy was defined in accordance with the Response Evaluation Criteria in Solid Tumors (RECIST) criteria.^24^ Serum tumor marker carbohydrate antigen 19.9 (CA19-9) was collected preoperatively and, if applicable, before neoadjuvant therapy. Serum CA19-9 level ≥37 U/mL was considered as elevated.

Extended resections were defined in accordance with the ISGPS definition.^20^ The type of PVR was defined following the ISGPS definition: tangential resection with primary closure (type 1), tangential resection with patch reconstruction (type 2), segmental resection with end-to-end reconstruction (type 3), and segmental resection reconstructed by using an interposition graft (type 4).^7^ Major morbidity was defined as Clavien-Dindo grade ≥IIIa^25^ and clinically relevant pancreatic surgery-specific complications [ie, delayed gastric emptying (DGE), postpancreatectomy hemorrhage (PPH), postoperative pancreatic fistula (POPF), and bile leak] were defined as grades B/C.^26–29^ Organ failure was defined as >24 hours need for mechanic respiratory support, dialyses, and/or inotropics.

Microscopic radicality was classified following either the 0 mm rule (ie, R0: >0 mm margin; R1: direct margin involvement), 1 mm rule (ie, R0: ≥1 mm margin; R1: <1 mm margin clearance), or the variant 1 mm rule (ie, R0: ≥1 mm margin; R1: <1 mm margin clearance; R1: direct margin involvement).^30^ In case of disease recurrence, the first location of disease recurrence was registered (ie, locoregional, distant, or both). Locoregional disease recurrence was defined as suspected recurrence at the operation site and/or locoregional lymphadenopathy. The diagnosis of disease recurrence was based on cross-sectional imaging and eventually pathology-proven. The RFS and OS were measured from the time of surgery since the findings from this study should facilitate clinical decision-making at the time of surgery.

### Statistical Analyses

Data analyses were performed by statistician M.A., using R, version 4.3.2 (R Foundation for Statistical Computing), and Stata version 17 (StataCorp). Statistical significance was considered as a 2-tailed *P*-value of <0.050.

Categorical variables are presented as percentages and frequencies, compared using the Pearson χ^2^ test. Continuous variables are presented as medians with interquartile ranges (IQR) and compared with the Mann-Whitney *U* test. The reverse Kaplan-Meier method was used to calculate the median follow-up. Unadjusted OS was estimated by the Kaplan-Meier method, measured from the date of surgery. Differences between PVR type 1–2 and type 3–4 were assessed with the log-rank test.

Binomial logistic regression analysis was performed to investigate the impact of PVR types 1–2 versus types 3–4 on major morbidity, adjusted for the following prespecified confounders known during surgery: age, body mass index (BMI), and ECOG performance status at time of surgery, neoadjuvant therapy versus upfront surgery, Cattell-Braasch maneuver,^31^ splenic vein preservation versus ligation (± reconstruction), and the center in which surgery took place. The results were presented as odds ratios (OR) with 95% CI. Interactions between the type of PVR and, respectively, neoadjuvant therapy and performance status were analyzed. Interactions were tested in multivariable logistic regression models for major morbidity adjusted for the covariates as used in the main multivariable logistic regression model. A likelihood ratio test was used to calculate the *P* value of each interaction.

Cox regression analysis was used to investigate the difference in RFS and OS between PVR types 1 to 2 versus types 3 to 4, adjusted for prespecified confounders known at time of surgery: age, sex, and ECOG performance status at time of surgery, tumor size, lymphadenopathy, and portomesenteric venous contour irregularity on cross-sectional imaging at diagnosis, neoadjuvant therapy versus upfront surgery, preoperative serum CA19-9,^32^ and the center in which surgery took place. Serum CA19-9 was transformed into a logarithmic scale. All continuous variables were modeled using restricted cubic splines with three knots to capture any nonlinear covariate-outcome relationships. Logistic regression was used to investigate the impact of the PVR type on locoregional recurrence, adjusted for the same prespecified confounders as for the RFS and OS regression analyses.

The proportional hazards assumption was checked using visual inspection of Schoenfeld residuals and the Grambsch-Therneau test. Results were presented as hazard ratios (HR) with 95% CI. Interactions between the type of PVR and respectively neoadjuvant therapy, portomesenteric venous contour irregularity on cross-sectional imaging, and performance status were analyzed. Interactions were tested in multivariable Cox regression models for OS adjusted for the covariates as used in the main multivariable Cox proportional hazards model. A likelihood ratio test was used to calculate the *P* value of each interaction.

Multiple imputation was used to account for data missing at random in the multivariable Cox and logistic regression analyses. The imputation model was congenial with the main analysis model, and included the treatment variable, all confounders in the Cox and logistic regression models, the event indicator (ie, death or censored), the Nelson-Aalen estimate of the baseline hazard, and whether a patient experienced major morbidity (ie, the outcome in the logistic regression model). In total, 50 imputed datasets (excluding 50 burn-in iterations) were created, and parameter estimates were pooled across imputed datasets using the Rubin rules. In addition, flexible parametric survival models and regression standardization were used to estimate adjusted absolute survival probabilities and median survival times for patients treated with PVR type 1 to 2 versus 3 to 4.^33–35^

## RESULTS

Overall, 357 patients with pancreatic cancer who underwent PVR during pancreatoduodenectomy were included, including 150 tangential (42%) and 207 segmental (58%) venous resections. Patients were treated in 10 centers including the Netherlands (3 centers: 98 patients), Sweden (3 centers: 89 patients), Japan (2 centers: 130 patients), and the United States (2 centers: 40 patients). See Appendix 1, Supplemental Digital Content 1, http://links.lww.com/SLA/F386 for the distribution of PVR types per center.

### Baseline Characteristics

Patients who underwent tangential PVR had a higher rate of ECOG PS ≥1 compared with patients treated with segmental PVR (51% vs 15%; *P*<0.0001). Tumor size on cross-sectional imaging at diagnosis was similar [median, 25 mm (IQR, 20–30) vs 25 (IQR, 20–31); *P*=0.24] between both groups, as was the presence of venous contour irregularity (19% *v*s 18%; *P*=0.90). Neoadjuvant therapy was administered in 23% and 21% of patients (*P*=0.76), respectively, in the tangential and segmental groups. Median preoperative serum CA19-9 level in the tangential group was 111 U/mL (IQR, 40–364) compared with 108 U/mL (IQR, 22–537) (*P*=0.95) in the segmental group. See Table 1 for preoperative clinicopathologic and treatment characteristics.

**TABLE 1:** Preoperative Clinical and Pathologic Characteristics

Variables	Tangential PVR (*n*=150)	Segmental PVR (*n*=207)	*P*
Baseline characteristics			
Age (median, IQR) (y)	71 (64 to 76)	69 (62 to 74)	0.13
Female sex, n (%)	75 (50)	93 (45)	0.40
BMI (median, IQR) (kg/m^2^)	24 (22 to 27)	22 (20 to 25)	**<0.0001**
Missing, n (%)	0 (0)	1 (<1)	
ECOG-PS, n (%)			**<0.0001**
ECOG-PS 0	58 (39)	166 (80)	
ECOG-PS ≥1	76 (51)	32 (15)	
Missing	16 (11)	9 (4)	
Disease characteristics at diagnosis			
Tumor size (median, IQR) (mm)	25 (20 to 30)	25 (20 to 31)	0.24
Missing, n (%)	7 (5)	5 (2)	
Clinical lymph node status, n (%)			0.44
cN0	125 (83)	181 (87)	
cN1-2	23 (15)	25 (12)	
Missing	2 (1)	1 (<1)	
Portomesenteric venous contour irregularity, n (%)	29 (19)	38 (18)	0.90
Missing, n (%)	1 (<1)	0 (0)	
Serum CA19-9 (median, IQR) (U/mL)	181 (46 to 436)	176 (33 to 758)	0.62
Missing, n (%)	16 (11)	12 (6)	
Neoadjuvant therapy			
Neoadjuvant therapy, n (%)	34 (23)	43 (21)	0.76
(m)FOLFIRINOX	17 (50)	12 (28)	**0.0005**
Other multi-agent chemotherapy	5 (15)	25 (58)	
Single-agent chemotherapy	12 (35)	6 (14)	
Second-line chemotherapy, n (%)	2 (6)	2 (5)	>0.99
Radiotherapy, n (%)	19 (13)*	12 (6)	**0.035**
Conventional radiotherapy	9 (47)	6 (50)	>0.99
SBRT	10 (53)	6 (50)	
Disease characteristics following neoadjuvant therapy			
RECIST, n (%)			0.99
Stable disease	25 (74)	32 (74)	
Partial/complete response	6 (18)	8 (19)	
Progressive disease	2 (6)	3 (7)	
Unknown	1 (3)	0 (0)	
Serum CA19-9 (median, IQR) (U/ml)	55 (31 to 107)	32 (9 to 71)	0.052
Missing, n (%)	4 (12)	2 (5)	

Bold value indicates statistical significance (*P*<0.050).

* n=1 is missing on whether preoperative radiation was given.

CA19-9 indicates carbohydrate antigen 19-9; ECOG-PS, Eastern Cooperative Oncology Group–Performance Status; IQR, interquartile range; n, number of patients; (m)FOLFIRINOX, (modified) combination of 5-fluorouracil with leucovorin, irinotecan, and oxaliplatin; PVR, portomesenteric venous resection; RECIST, Response Evaluation in Solid Tumours; SBRT, stereotactic body radiation therapy.

### Surgical Procedures and Outcome

In the group of tangential PVR, operation time [median, 419 (IQR, 333–485) vs 494 (IQR, 442–566); *P*<0.0001] and intraoperative blood loss [median, 500 (IQR, 300–750) vs 600 (IQR, 350–938); *P*=0.020] were lower compared with segmental PVR. The use of any type of graft to reconstruct the portomesenteric vein was higher in patients who underwent tangential compared with segmental PVR (10% vs 3%; *P*=0.005). The splenic vein was preserved during tangential PVR in 95% of patients, whereas the splenic vein was resected during segmental PVR in 50% of patients (*P*<0.0001), in whom the splenic vein was reconstructed in 42% of patients (n=43/103).

The rates of in-hospital/30-day major morbidity (29% vs 21%; *P*=0.10) and 90-day mortality (4% vs 4%; *P*=0.95) did not differ significantly between patients undergoing tangential and segmental PVR. No differences were observed in the rates of PPH grade B/C (6% vs 6%; *P*>0.99) and portomesenteric venous thrombosis (6% vs 7%; *P*=0.77). The median postoperative hospital stay was shorter after tangential compared with segmental PVR [median, 10 (IQR, 8–15) vs 19 (IQR, 12–30); *P*<0.0001]. See Table 2 for details about the surgery and surgical outcome and Appendix 2, Supplemental Digital Content 1, http://links.lww.com/SLA/F386 for surgery and surgical outcome in patients who underwent segmental PVR, stratified for splenic vein ligation and splenic vein reconstruction.

**TABLE 2:** Surgical Procedure and Surgical Outcome

Variables	Tangential PVR (n=150)	Segmental PVR (n=207)	*P*
Surgical procedures			
Surgical modality, n (%)			**0.016**
Open	141 (94)	205 (99)	
Minimally invasive	9 (6)	2 (<1)	
Conversion to open	1 (11)	1 (50)	
Operation time (median, IQR) (min)	419 (333–485)	494 (442–566)	**<0.0001**
Missing, n (%)	25 (17)	12 (6)	
Intraoperative blood loss (median, IQR) (mL)	500 (300–750)	600 (350–938)	**0.020**
Missing, n (%)	5 (3)	5 (2)	
Intraoperative blood transfusion(s), n (%)	14 (9)	24 (12)	0.59
Missing	2 (1)	5 (2)	
Cattell-Braasch maneuver, n (%)	24 (16)	52 (25)	0.054
Missing	3 (2)	3 (1)	
Graft, n (%)*	15 (10)	6 (3)	**0.005**
Autologous	9 (60)	4 (67)	
Vein	3 (33)	4 (100)	
Peritoneum/falciform ligament	5 (56)	0 (0)	
Other	1 (11)	0 (0)	
Biological graft	6 (40)	1 (17)	
Prosthetic graft	0 (0)	1 (17)	
Splenic vein, n (%)			**<0.0001**
Preservation	143 (95)	99 (48)	
Resection	4 (3)	103 (50)	
No reconstruction	4 (100)	60 (58)	
Reconstruction	0 (0)	43 (42)	
Reinsertion in portomesenteric axis	0 (0)	1 (2)	
Splenorenal shunt	0 (0)	37 (86)	
Other	0 (0)	5 (12)	
Missing	3 (2)	5 (2)	
In-hospital/30-day surgical outcome			
Major morbidity, n (%)	44 (29)	44 (21)	0.10
Relaparotomy, n (%)	2 (1)	11 (5)	0.090
Organ failure, n (%)	6 (4)	8 (4)	
Missing	0 (0)	1 (<1)	> 0.99
Delayed gastric emptying grade B/C, n (%)	30 (20)	27 (13)	0.10
Postoperative pancreatic fistula grade B/C, n (%)	8 (5)	20 (10)	0.19
Postpancreatectomy hemorrhage grade B/C, n (%)	9 (6)	12 (6)	> 0.99
Bile leakage grade B/C, n (%)	5 (3)	0 (0)	**0.029**
Portomesenteric venous thrombosis, n (%)	9 (6)	14 (7)	0.77
Postoperative hospital stay (median, IQR) (d)	10 (8–15)	19 (12–30)	**< 0.0001**
Missing, n (%)	0 (0)	1 (<1)	
Mortality, n (%)	1 (<1)	3 (1)	0.49

Bold value indicates statistical significance (*P*<0.050).

* Patients who underwent PVR type 2 or 4.

IQR indicates interquartile range; n, number of patients.

After adjusting for confounders, the type of PVR was not associated with major morbidity [OR=1.01 (95% CI, 0.54–1.90)] with an adjusted risk of 23% (95% CI, 17–32) after tangential and 23% (95% CI, 17–30) after segmental PVR. The adjusted risk difference was 0.15% (95% CI, −11 to 11%; *P*=0.98) lower after segmental PVR. See Table 3 for the logistic regression analysis on major morbidity.

**TABLE 3:** Logistic Regression Analysis–Major Morbidity

		Univariable analysis		Multivariable analysis	
Variables	n/total	OR (95% CI)	*P*	OR (95% CI)	*P*
Age at time of surgery (per 5 y increase)	357 (100)	1.03 (0.90–1.17)	0.67	1.03 (0.89–1.18)	0.71
BMI at time of surgery (per 5 kg/m^2^ increase)	356 (100)	1.14 (0.85–1.54)	0.38	1.11 (0.80–1.54)	0.53
ECOG-PS at the time of surgery			0.23		0.74
ECOG-PS 0	224 (63)	[Referent]		[Referent]	
ECOG-PS ≥1	108 (30)	1.36 (0.80–2.31)		0.96 (0.50–1.86)	
Neoadjuvant therapy			0.55		0.52
Upfront surgery	280 (78)	[Referent]		[Referent]	
Neoadjuvant therapy	77 (22)	0.83 (0.46–1.52)		0.81 (0.43–1.54)	
Cattell-Braasch maneuver			0.50		0.42
No	275 (77)	[Referent]		[Referent]	
Yes	76 (21)	0.82 (0.44–1.51)		0.74 (0.37–1.51)	
Splenic vein			**0.005**		**0.030**
Resection with reconstruction	43 (12)	[Referent]		[Referent]	
Resection without reconstruction	64 (18)	2.20 (0.66–7.35)		2.55 (0.72–8.98)	
Preservation	242 (68)	3.93 (1.35–11.4)		4.00 (1.26–12.6)	
PVR type			0.082		0.92
Tangential (ie, type 1–2)	150 (42)	[Referent]		[Referent]	
Segmental (ie, type 3–4)	207 (58)	0.65 (0.40–1.06)		1.01 (0.54–1.90)	

Bold value indicates statistical significance (*P*<0.050).

ECOG-PS indicates Eastern Cooperative Oncology Group–Performance Status; n, number of patients; OR, odds ratio; PVR, portomesenteric venous resection.

The interaction analysis demonstrated that the absent association of PVR type with major morbidity was consistent among sub-groups, regarding neoadjuvant therapy versus upfront surgery (*P*_interaction_=0.89) and ECOG performance status 0 versus ≥1 (*P*_interaction_=0.77).

### Histopathology and Adjuvant Therapy

Tumor size did not differ significantly between the tangential and segmental groups [median, 30 (IQR, 25–38) vs 34 (IQR, 25–40); *P*=0.10]. The R1 rate was higher after tangential compared with segmental PVR (61% vs 40%; *P*<0.0001); including the portomesenteric venous (41% vs 24%; *P*=0.0009) and SMA (25% vs 14%; *P*=0.015) margins. See Appendix 3, Supplemental Digital Content 1, http://links.lww.com/SLA/F386 for more details about the specific resection margins.

The administration of adjuvant chemotherapy was nonsignificantly lower among patients after tangential compared with segmental resection (68% vs 76%; *P*=0.072), in which single-agent regimens were more often used in the segmental group (57% vs 75%; *P*=0.017). See Appendix 4, Supplemental Digital Content 1, http://links.lww.com/SLA/F386 for the details about histopathology and adjuvant therapy.

### Oncological Outcome

Overall, 259 patients (73%) died during a median follow-up of 23 months (IQR, 11–53). The median RFS after tangential resection was 13 months (95% CI, 11–19) compared with 15 months (95% CI, 12–18) after segmental resection (*P*=0.47). After adjusting for confounders, the type of PVR was not associated with RFS [HR=0.94 (95% CI, 0.69–1.28)]. The rates of locoregional recurrence (with or without synchronous distant metastases) did not differ significantly between tangential and segmental PVR (44% vs 36%; *P*=0.056). After adjusting for confounders, PVR type was not associated with locoregional recurrence [OR=0.76 (95% CI, 0.40–1.46)] with an adjusted risk of 43% (95% CI, 30–56) after tangential PVR and 37% (95% CI, 25–49) after segmental PVR [adjusted risk difference=6% (95% CI −8% to 20%); *P*=0.38].

**FIGURE 1:**
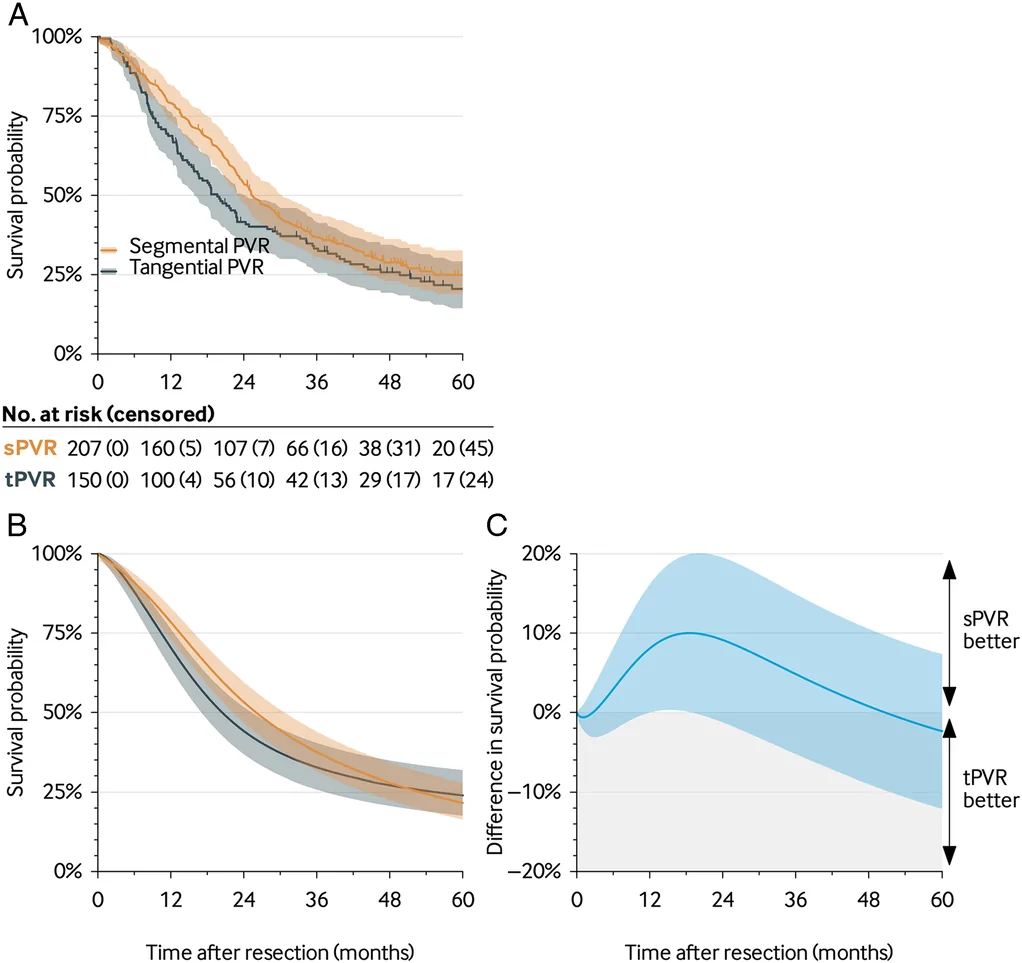
Overall survival–tangential versus segmental PVR.

Overall, the median OS was 23 months (IQR, 12–53), with a 1-, 3-, and 5-year OS of 75% (95% CI, 71–80), 35% (95% CI, 31–40), and 23% (95% CI, 19–29), respectively. Comparing tangential versus segmental PVR, the OS did not differ significantly considering a median and 5-year OS of 20 months (95% CI, 16–24) and 21% (95% CI, 14–29) versus 25 months (95% CI, 23–30), and 25% (95% CI, 19–33), respectively. After adjusting for confounders, segmental PVR was not associated with OS compared with tangential PVR [HR=0.94 (95% CI, 0.69–1.30)]. See Figure 1 for the survival curves, Table 4 for the Cox regression analysis, and Appendix 5, Supplemental Digital Content 1, http://links.lww.com/SLA/F386 for the association strength of the preoperative and intraoperative parameters with OS. The adjusted median OS after tangential and segmental PVR was 20 months (95% CI, 17–24) versus 25 months (95% CI, 22–30) (*P*=0.073) with 5-year OS rates of 24% (95% CI, 18–32) versus 22% (95% CI, 16–28) (*P*=0.63).

**TABLE 4:** Cox Regression Analysis–Overall Survival

		Univariable analysis		Multivariable analysis	
Variables	n/total	HR (95% CI)	*P*	HR (95% CI)	*P*
Age at time of surgery (per 5 y increase)	357 (100)	1.05 (0.98–1.12)	0.18	1.00 (0.93–1.07)	0.93
Sex			0.95		0.89
Female	168 (47)	[Referent]		[Referent]	
Male	189 (53)	0.99 (0.78–1.27)		1.01 (0.78–1.32)	
BMI at time of surgery (per 5 kg/m^2^ increase)	356 (100)	0.93 (0.80–1.09)	0.40	0.86 (0.73–1.03)	0.10
ECOG-PS at the time of surgery			**0.003**		0.091
ECOG-PS 0	224 (63)	[Referent]		[Referent]	
ECOG-PS ≥1	108 (30)	1.48 (1.14–1.92)		1.38 (0.96–1.96)	
Tumor size (per 10 mm increase)	345 (97)	1.07 (0.92–1.24)	0.36	0.97 (0.83–1.14)	0.74
Portomesenteric venous contour irregularity			0.37		0.39
No	289 (81)	[Referent]		[Referent]	
Yes	67 (19)	1.15 (0.85–1.57)		1.16 (0.83–1.61)	
Clinical lymph node status			0.55		0.51
cN0	306 (86)	[Referent]		[Referent]	
cN1-2	48 (14)	0.90 (0.62–1.30)		0.87 (0.59–1.31)	
Neoadjuvant therapy			0.25		0.62
Upfront surgery	280 (78)	[Referent]		[Referent]	
Neoadjuvant therapy	77 (22)	0.84 (0.61–1.14)		1.10 (0.77–1.55)	
Preoperative serum CA19-9			**<0.0001**		**<0.0001**
<37	89 (25)	[Referent]		[Referent]	
≥37–<150	93 (26)	1.43 (0.99–2.06)		1.45 (0.99–2.12)	
≥150–<500	73 (20)	1.87 (1.29–2.72)		1.81 (1.20–2.72)	
≥500	73 (20)	3.16 (2.19–4.55)		3.45 (2.28–5.21)	
PVR type			0.18		0.74
Tangential (ie, type 1–2)	150 (42)	[Referent]		[Referent]	
Segmental (ie, type 3–4)	207 (58)	0.84 (0.66–1.08)		0.94 (0.69–1.30)	

Bold value indicates statistical significance (*P*<0.050).

ECOG-PS indicates Eastern Cooperative Oncology Group–Performance Status; HR, hazard ratio; n, number of patients; PVR, portomesenteric venous resection.

The interaction analysis demonstrated that the absence of an association of PVR type with OS was consistent among subgroups regarding neoadjuvant therapy versus upfront surgery (*P*_interaction_=0.089), ECOG performance status 0 versus ≥1 (*P*_interaction_=0.38), PMV involvement with or without wall irregularity on cross-sectional imaging at diagnosis (*P*_interaction_=0.63), and preoperative serum CA19-9 (*P*_interaction_=0.90).

## DISCUSSION

This international retrospective multicenter study, including 357 patients who underwent PVR during pancreatoduodenectomy for pancreatic cancer with only ≤180 degrees PMV involvement, demonstrated that segmental PVR is not associated with OS [adjusted median OS of 25 vs 20​​​​​​ ​mo (*P*=0.073) with a 5-y OS rate of 22% vs 24% (*P*=0.63)] compared with tangential PVR. Moreover, segmental PVR is not associated with the locoregional recurrence rate (adjusted risk of 37% vs 43%; *P*=0.38) compared with tangential PVR, despite a higher R1 rate (40% vs 61%; *P*<0.0001) after tangential PVR. The type of PVR was not associated with major morbidity after adjusting for confounders (23% vs 23%; *P*=0.98), including similar rates of portomesenteric venous thrombosis after segmental and tangential PVR (7% vs 6%; *P*=0.77).

A previous multicenter observational study compared tangential versus segmental PVR among 177 patients with pancreatic cancer who underwent any type of pancreatic resection.^11^ After propensity score matching (n=53 vs n=53), 90-day major morbidity did not differ significantly between tangential and segmental PVR (25% vs 38%; *P*=0.100). However, the multivariable Cox regression analysis showed that segmental PVR was associated with impaired disease-specific survival, but without significant differences in R1 (63% vs 63%; *P*=0.500) and locoregional recurrence (27% vs 18%; *P*=0.580) rates between tangential and segmental PVR.^11^ These findings conflict with our study in which no association was found between the type of PVR and OS in a more homogenous cohort of patients with limited venous involvement and without arterial involvement, in which both types of PVR were assumed to be possible due to limited venous involvement. Moreover, the selection in the present study might also have been reduced by the different preferences between centers for either tangential or segmental PVR (Appendix 1, Supplemental Digital Content 1, http://links.lww.com/SLA/F386). Of note, the preference for either tangential or segmental PVR within more than half of the participating centers could be the reason that the unadjusted length of hospital stay was almost twice as long after segmental compared with tangential PVR in this study, as this might be influenced by local protocols.

In the current study, the higher R1 rate after tangential PVR compared with segmental PVR—particularly driven by the portomesenteric venous resection margin—could also have been explained by a “*false”* R1 from the portomesenteric venous margin due to ex vivo shrinkage from the smaller venous patch due to a less wide resection margin when performing a tangential PVR compared with segmental PVR. Hence, the higher rate of R1 did not result into an increased risk for locoregional recurrence in this study (adjusted risk of 43% vs 37%; *P*=0.38). On the other hand, the difference in R-status in this study should be interpreted with caution as these outcomes can be influenced by differences in local pathology protocols with regard to the definition of R0/R1 (ie, 1 mm rule, 1 mm variant rule, 0 mm rule) and which PMV margins were assessed (ie, venous transection margin, venous circumferential margin, portomesenteric venous groove). A single-center observational study including 81 patients with pT3 pancreatic head cancer who underwent a pancreatic resection with PVR showed that R-status was not associated with OS.^36^ Possibly, this is caused by the actual higher rate of R1 than reported due to invasion in the portomesenteric venous groove in up to 92% of venous resections, as demonstrated by Verbeke and colleagues.^37^

The results from our study suggest that pancreatic cancer located in the pancreatic head with limited PMV involvement can be managed with either a tangential or segmental PVR, considering the similar surgical and oncological outcome. The decision should be driven based on the surgeon’s individual preference and experience. Some surgeons advocate for a segmental PVR as it may reduce the risk of angulation or narrowing of the reconstruction, thereby reducing the risk of thrombosis. Others prefer a tangential PVR for these right-sided tumors so that the splenic vein can be preserved, reducing the risk for left-sided portal hypertension,^38^ even though the splenic vein could be reconstructed.^39,40^ In the current study, no significant difference was observed in the incidence of PMV thrombosis between segmental and tangential PVR (7% vs 6%). Of note, this could be influenced by potential differences in use and type of perioperative thromboprophylaxis between centers, wherefore similar rates should be interpreted with caution. The information on perioperative thromboprophylaxis was not collected considering the limited reliability related to the retrospective data collection. The 6% to 7% in-hospital/30-day PMV thrombosis rate is lower compared with the international in-hospital benchmark of ≤14%.^41^ Possibly, this is caused by the limited use of synthetic graft material in this study (<1%), as synthetic grafts are associated with an increased risk for thrombosis.^42^ In the case of a segmental PVR, the need for an interposition graft can be reduced with a Cattell-Braasch maneuver.^31,43^ See Appendix 6, Supplemental Digital Content 1, http://links.lww.com/SLA/F386 for the difference in surgical and oncological outcomes between the international benchmarks for pancreatoduodenectomy with PVR compared with the current study results.^41^

The results from this study should be interpreted in the light of several limitations. First, venous involvement was based on cross-sectional imaging at diagnosis instead of intraoperative assessment, since this was considered the only feasible and reliable strategy for this retrospective study. Second, no central review of cross-sectional imaging was performed and no established maximum time interval between imaging and surgery was established. Therefore, it cannot be excluded that the vascular involvement progressed to some extent before surgery, which may be illustrated by the occurrence of R1 on the SMA margin. Moreover, there could be infiltration of the PMV caused by pancreatitis following endoscopic retrograde cholangiopancreatography, which could have influenced the choice for either a tangential or segmental PVR. This illustrates the risk for residual confounding as information about intraoperative PMV involvement/infiltration and decision-making is impossible to extract retrospectively. Third, no information was collected about the precise level (ie, portal vein, confluence, superior mesenteric vein) and side (right or left) of PMV involvement, considering the retrospective design of this study.^44^ Nevertheless, the major strength of this series is its homogeneity by including solely patients where both a tangential and segmental PVR were assumed to be technically possible. Moreover, adjustments were made for only factors known at the time of surgery, whereby the current results can be used for intraoperative decision-making.

Future studies should investigate whether patients with resectable pancreatic cancer with ≤180 degrees PMV involvement benefit from neoadjuvant therapy compared with upfront surgery.^45,46^ Moreover, the surgical safety and oncological outcome of tangential and segmental PVR should be studied separately for patients with a tumor located in the pancreatic body.^47,48^ The risk of clinically relevant sinistral portal hypertension after splenic vein resection with or without reconstruction should be further investigated.^38^ Furthermore, the value of thromboprophylaxis, including different strategies, has to be clarified in patients undergoing PVR^49,50^ and pathology protocols should be standardized with regard to the assessment of pathologic and surgical margins. At last, only a randomized controlled trial with intraoperative randomization can give a definite answer on the oncological outcome between tangential and segmental PVR in patients with pancreatic head cancer and ≤180 degrees PMV involvement undergoing pancreatoduodenectomy.

## CONCLUSION

In patients with pancreatic head cancer and ≤180 degrees PMV involvement undergoing pancreatoduodenectomy, segmental PVR can be performed as safely as tangential PVR without impacting recurrence and OS. This suggests that if both procedures are technically feasible, surgeons may decide between segmental and tangential PVR depending on their personal experience and preference.

## Supplementary Material
