# Supplementary material for: Tangential Versus Segmental Portomesenteric Venous Resection During Pancreatoduodenectomy for Pancreatic Cancer: An International Multicenter Cohort Study on Surgical and Oncological Outcome
Source: Ann Surg. 2025 Jan 22;284(4):821–30. doi: 10.1097/SLA.0000000000006638 (PMC13557467; doi:10.1097/SLA.0000000000006638)
Supplement: Supplementary file 1 [file sla-284-821-s001.docx]

**APPENDIX 1.** Types of portomesenteric venous resections per center

Percentage of tangential (tPVR) and segmental (sPVR) portomesenteric venous resection per center. Centers are sorted by the percentage of tangential PVRs that are performed.

For each center, the **blue dot** represents the percentage of tangential PVR performed from the total number of included patients from that center, whereas the **orange dot** represents the percentage of segmental PVR from the total number of included patients from that center.

| **APPENDIX 2**. Surgical procedure and outcome after segmental PVR – Impact of splenic vein resection and reconstruction | | | | | | | | | | | | |
| --- | --- | --- | --- | --- | --- | --- | --- | --- | --- | --- | --- | --- |
| **Variables** | **Splenic vein preservation** | | **Splenic vein resection** | | | | | | **P value*** | | **P value**** | |
|  |  | | **Overall** | | **No reconstruction** | | **Reconstruction** | |  |  |  |  |
| **Patients** | **99** | | **103** | | **60** | | **43** | |  | |  | |
| **Surgical procedures** | | | | | | | | | | | | |
| Operation time (median, IQR) (min) | 475 | (394 to 555) | 500 | (461 to 574) | 503 | (451 to 602) | 500 | (484 to 551) |  | **0.005** |  | 0.90 |
| Missing, *n* (%) | 8 | (8) | 4 | (4) | 4 | (7) | 0 | (0) |  |  |  |  |
| Intraoperative blood loss (median, IQR) (ml) | 600 | (355 to 923) | 590 | (335 to 945) | 600 | (324 to 1050) | 520 | (340 to 790) |  | 0.42 |  | 0.26 |
| Missing, *n* (%) | 3 | (3) | 2 | (2) | 2 | (3) | 0 | (0) |  |  |  |  |
| Intraoperative blood transfusion(s), *n* (%) | 14 | (14) | 8 | (8) | 8 | (13) | 0 | (0) |  | 0.13 |  | **0.012** |
| Missing | 4 | (4) | 1 | (<1) | 1 | (2) | 0 | (0) |  |  |  |  |
| Cattell-Braasch maneuver, *n* (%) | 28 | (28) | 23 | (22) | 22 | (37) | 1 | (2) |  | 0.27 | < | **0.001** |
| Missing | 3 | (3) | 0 | (0) | 0 | (0) | 0 | (0) |  |  |  |  |
| **In-hospital/30-day surgical outcome** | | | | | | | | | | | | |
| Major morbidity, *n* (%) | 28 | (28) | 15 | (15) | 11 | (18) | 4 | (9) |  | **0.017** |  | 0.20 |
| Relaparotomy, *n* (%) | 5 | (5) | 5 | (5) | 4 | (7) | 1 | (2) |  | 0.95 |  | 0.31 |
| Organ failure, *n* (%)  Missing | 6  0 | (6)  (0) | 1  1 | (<1)  (<1) | 1  1 | (2)  (2) | 0  0 | (0)  (0) |  | 0.050 |  | 0.39 |
| Delayed gastric emptying grade B/C, *n* (%) | 15 | (15) | 12 | (12) | 8 | (13) | 4 | (9) |  | 0.47 |  | 0.53 |
| Postoperative pancreatic fistula grade B/C, *n* (%) | 13 | (13) | 7 | (7) | 5 | (8) | 2 | (5) |  | 0.13 |  | 0.46 |
| Postpancreatectomy hemorrhage grade B/C, *n* (%) | 8 | (8) | 4 | (4) | 3 | (5) | 1 | (2) |  | 0.21 |  | 0.49 |
| Bile leakage grade B/C, *n* (%) | 0 | (0) | 0 | (0) | 0 | (0) | 0 | (0) |  | NE |  | NE |
| Portomesenteric venous thrombosis, *n* (%) | 5 | (5) | 8 | (8) | 8 | (13) | 0 | (0) |  | 0.43 |  | **0.013** |
| Postoperative hospital stay (median, IQR) (days) | 18 | (11 to 30) | 20 | (14 to 28) | 15 | (9 to 25) | 22 | (19 to 33) |  | 0.43 | **<** | **0.001** |
| Missing, *n* (%) | 0 | (0) | 1 | (<1) | 1 | (2) | 0 | (0) |  |  |  |  |
| Mortality, *n* (%) | 3 | (3) | 0 | (0) | 0 | (0) | 0 | (0) |  | 0.075 |  | NE |
| **Patients with missing data on splenic vein ligation are excluded.** *n*, number of patients; *min*, minutes; *IQR*, interquartile range; *ml*, milliliters; *mm*, millimeter; *NE,* not estimable. Bold value indicates statistical significance (P<0.050). *Splenic vein preservation versus splenic vein resection (± reconstruction). **Splenic vein resection without reconstruction versus splenic vein resection with reconstruction. | | | | | | | | | | | | |

| **APPENDIX 3**. Resection margins | | | | | | |  |
| --- | --- | --- | --- | --- | --- | --- | --- |
| **Variables** | **Tangential PVR**  (n=150) | | **Segmental PVR**  (n=207) | | **P value** | | |
| Radicality status – Any definition, *n* (%) |  |  |  |  |  |  | |
| R0 | 57 | (38) | 125 | (60) |  |  | |
| R1 | 92 | (61) | 82 | (40) |  |  | |
| *Portomesenteric vein* | 61 | (41) | 50 | (24) |  |  | |
| *Superior mesenteric artery* | 37 | (25) | 30 | (14) |  |  | |
| *Pancreatic neck/body* | 9 | (6) | 13 | (6) |  |  | |
| *Common bile duct* | 5 | (3) | 0 | (0) |  |  | |
| *Stomach / proximal duodenum* | 3 | (2) | 1 | (<1) |  |  | |
| *Distal duodenum / jejunum* | 3 | (2) | 1 | (<1) |  |  | |
| *Posterior pancreatic surface* | 13 | (9) | 10 | (5) |  |  | |
| *Anterior pancreatic surface* | 10 | (9) | 12 | (6) |  |  | |
| *Other margin(s)* | 1 | (<1) | 3 | (1) |  |  | |
| Missing | 1 | (<1) | 0 | (0) |  |  | |
| Residual disease – 0mm rule, *n* (%) |  |  |  |  |  | 0.30 | |
| R0 | 10 | (7) | 103 | (50) |  |  | |
| R1 | 3 | (2) | 15 | (7) |  |  | |
| Residual disease – 1mm rule, *n* (%) |  |  |  |  |  | 0.16 | |
| R0 | 39 | (26) | 19 | (9) |  |  | |
| R1 | 74 | (49) | 57 | (28) |  |  | |
| Residual disease – Variant 1mm rule, *n* (%) |  |  |  |  |  | 0.46 | |
| R0 | 8 | (5) | 3 | (1) |  |  | |
| R1 | 15 | (10) | 10 | (5) |  |  | |
| *n*, number of patients. Bold value indicates statistical significance (P<0.050). | | | | | | |  |

| **APPENDIX 4**. Histopathology, adjuvant therapy, and recurrence | | | | | |  |
| --- | --- | --- | --- | --- | --- | --- |
| **Variables** | **Tangential PVR**  (n=150) | | **Segmental PVR**  (n=207) | | **P value** |  |
| **Histopathology** |  |  |  |  |  |  |
| Tumor size (median, IQR) (mm) | 30 | (25 to 38) | 34 | (25 to 40) | 0.10 |  |
| Missing, *n* (%) | 4 | (3) | 1 | (<1) |  |  |
| Lymph nodes, *n* (%) |  |  |  |  | 0.37 |  |
| (y)pN0 | 37 | (25) | 52 | (25) |  |  |
| (y)pN1 | 66 | (44) | 75 | (36) |  |  |
| (y)pN2 | 47 | (31) | 80 | (39) |  |  |
| Radicality status – Any definition, *n* (%) |  |  |  |  | **<0.0001** |  |
| R0 | 57 | (38) | 125 | (60) |  |  |
| R1 | 92 | (61) | 82 | (40) |  |  |
| *Portomesenteric vein* | 61 | (41) | 50 | (24) |  |  |
| *Superior mesenteric artery* | 37 | (25) | 30 | (14) |  |  |
| Missing | 1 | (<1) | 0 | (0) |  |  |
| Perineural invasion, *n* (%) | 131 | (87) | 195 | (94) | 0.12 |  |
| Unknown | 5 | (3) | 1 | (<1) |  |  |
| Lymphovascular invasion, *n* (%) | 101 | (67) | 163 | (79) | 0.069 |  |
| Unknown | 12 | (8) | 7 | (3) |  |  |
| Tumor grade, *n* (%) |  |  |  |  | 0.067 |  |
| Gx - Not possible to determine | 4 | (3) | 5 | (2) |  |  |
| G1 - Well differentiated | 10 | (7) | 27 | (13) |  |  |
| G2 - Moderately differentiated | 79 | (53) | 112 | (54) |  |  |
| G3 - Poorly differentiated | 46 | (31) | 42 | (20) |  |  |
| Missing | 11 | (7) | 21 | (10) |  |  |
| **Adjuvant therapy** |  |  |  |  |  |  |
| Adjuvant chemotherapy, *n* (%) | 102 | (68) | 158 | (76) | 0.072 |  |
| (m)FOLFIRINOX | 10 | (10) | 12 | (8) | **0.017** |  |
| Other multi-agent regimen | 29 | (28) | 25 | (16) |  |  |
| Single-agent chemotherapy | 58 | (57) | 119 | (75) |  |  |
| Unknown | 5 | (5) | 2 | (1) |  |  |
| Unknown | 2 | (1) | 3 | (1) |  |  |
| **Recurrence** |  |  |  |  |  |  |
| Recurrence, *n* (%) | 100 | (67) | 141 | (68) | 0.57 |  |
| Locoregional | 31 | (31) | 41 | (29) | 0.12 |  |
| Locoregional + distant | 35 | (35) | 34 | (24) |  |  |
| Distant  Unknown | 34  0 | (34)  (0) | 64  2 | (45)  (1) |  |  |
| Unknown | 11 | (7) | 3 | (1) |  |  |
| *n*, number of patients; *IQR*, interquartile range; Bold value indicates statistical significance (P<0.050). | | | | | |  |

**APPENDIX 5.** Strength of associations from pre- and intraoperative parameters with major morbidity and overall survival

| **APPENDIX 6**. International benchmarks versus present study | | | | | | | |
| --- | --- | --- | --- | --- | --- | --- | --- |
| **Variables** | **Present study** | | **International benchmarks^43^** | | Δ | | |
| **Primary outcome** | | | | | | | |
| Overall survival |  |  | |  | |  |  |
| 3-year |  |  | | ≥21% | |  |  |
| Tangential PVR | 33 | (26 to 42) | |  | | 12 | (5 to 21) |
| Segmental PVR | 37 | (31 to 45) | |  | | 16 | (10 to 24) |
| 5-year |  |  | | ≥9% | |  |  |
| Tangential PVR | 21 | (14 to 29) | |  | | 12 | (5 to 20) |
| Segmental PVR | 25 | (19 to 33) | |  | | 16 | (10 to 24) |
| **Secondary outcomes** | | | | | | | |
| In-hospital/30-day major morbidity* |  |  | | ≤28% | |  |  |
| Tangential PVR | 29 | (23 to 37) | |  | | +1 | (–5 to 9) |
| Segmental PVR | 21 | (16 to 27) | |  | | –7 | (–12 to –1) |
| In-hospital mortality/30-day mortality* |  |  | | ≤4% | |  |  |
| Tangential PVR | 4 | (2 to 8) | |  | | 0 | (–2 to 4) |
| Segmental PVR | 4 | (2 to 7) | |  | | 0 | (–2 to 3) |
| In-hospital/30-day portomesenteric venous thrombosis* |  |  | | ≤14% | |  |  |
| Tangential PVR | 6 | (3 to 11) | |  | | –8 | (–11 to –3) |
| Segmental PVR | 7 | (4 to 11) | |  | | –7 | (–10 to –3) |
| R1 |  |  | | ≤63% | |  |  |
| Tangential PVR | 62 | (54 to 69) | |  | | –1 | (–9 to 6) |
| Segmental PVR | 40 | (33 to 46) | |  | | –23 | (–30 to –17) |
| *In-hospital major morbidity and mortality was used by Raptis and colleagues. | | | | | | | |
